# Supplementary material for: Functional genetic variants can mediate their regulatory effects through alteration of transcription factor binding
Source: Nat Commun. 2019 Aug 2;10:3472. doi: 10.1038/s41467-019-11412-5 (PMC6677801; doi:10.1038/s41467-019-11412-5)
Supplement: Supplementary file 1 — Supplementary Information [file 41467_2019_11412_MOESM1_ESM.pdf]

FUNCTIONAL GENETIC VARIANTS MEDIATE THEIR REGULATORY EFFECTS THROUGH ALTERED TRANSCRIPTION FACTOR BINDING.

Andrew D. Johnston, Claudia A. Simões-Pires, Taylor V. Thompson, Masako Suzuki, John M. Greally

SUPPLEMENTARY FIGURES

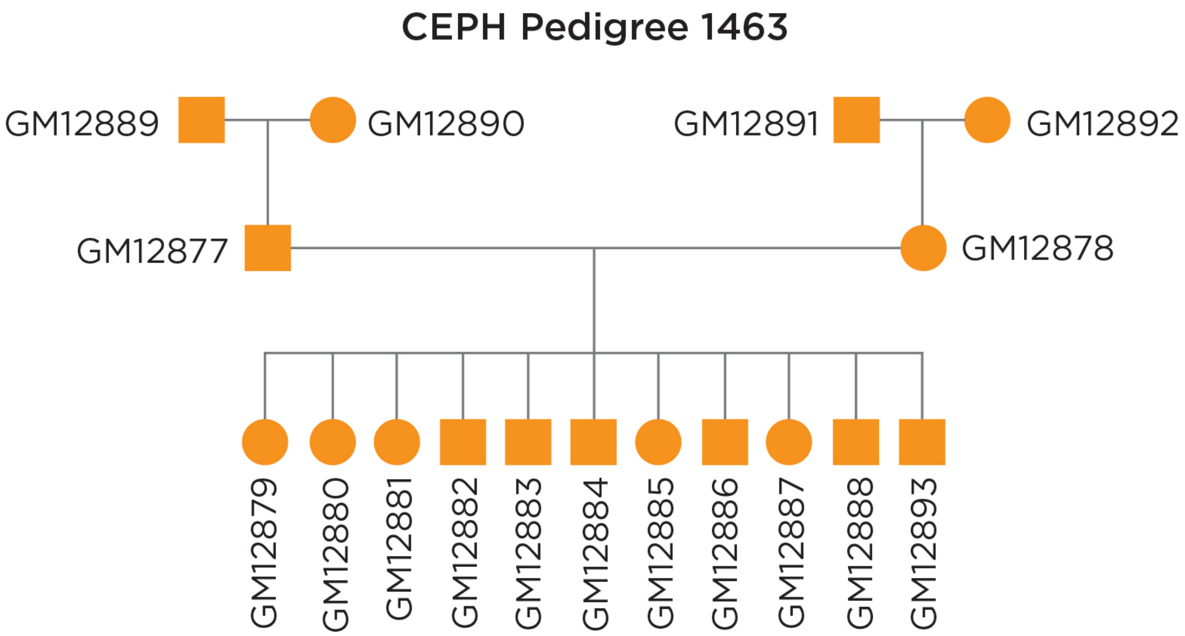

Figure S1: The 17 member family on whose LCLs we performed functional assays.

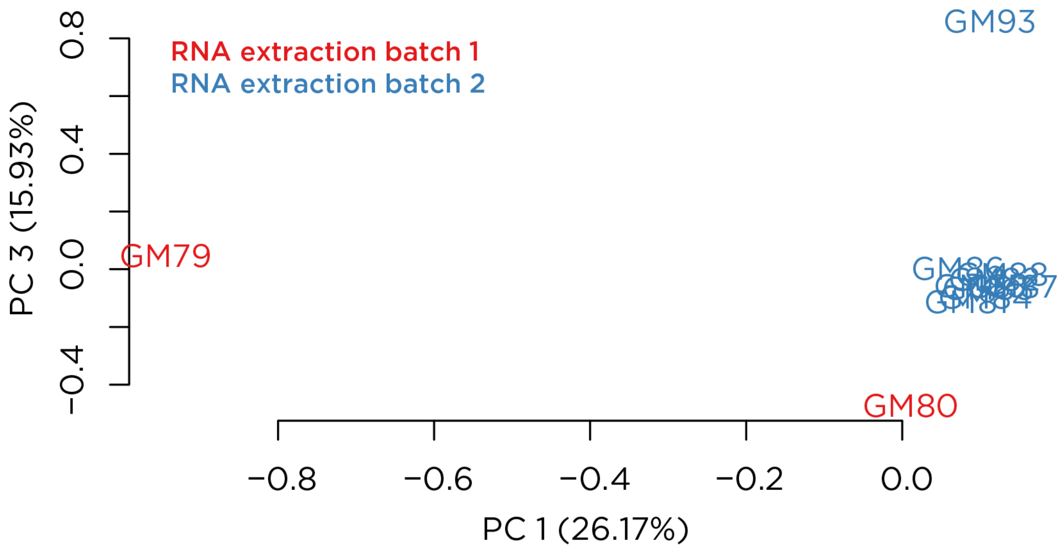

Figure S2: Principal components analysis of samples (key: “GM79” shorthand for GM12879) following removal of sex chromosome and EBV genes. The blue and red colors indicate two extraction dates, in which GM12879 and GM12880 differed from the other samples, revealing a likely influence for that component of the sample processing on gene expression variation. The reason for GM12893 to have distinctive expression is addressed in **Figure S3** below.

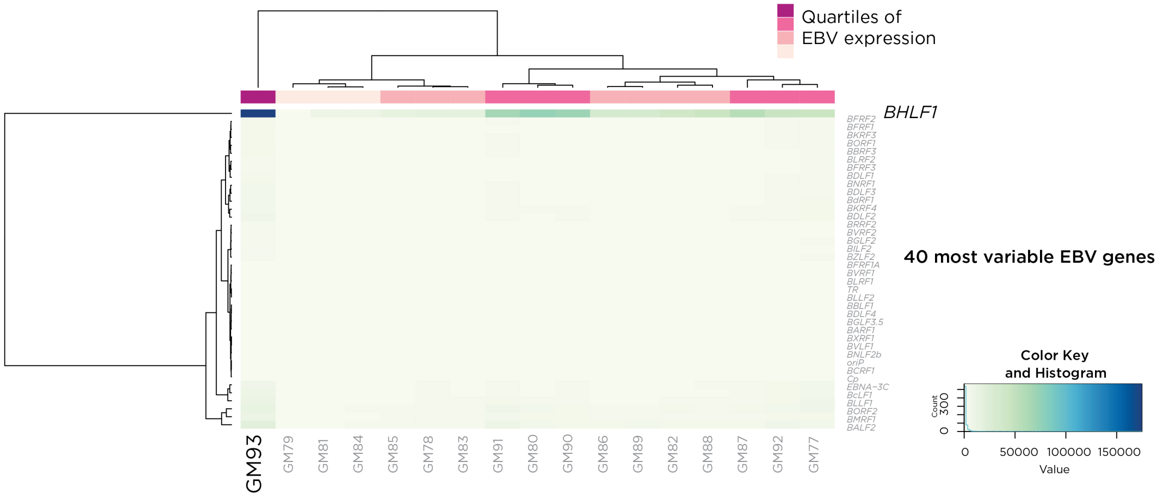

Figure S3: The expression levels of the 40 most variably expressed EBV genes are depicted in the heat map above. The *BHLF1* gene is distinctively over-expressed in GM12893. As this gene is characteristically expressed in the lytic phase of EBV infection, this is likely to explain the variation of expression of this sample shown in **Figure S2**.

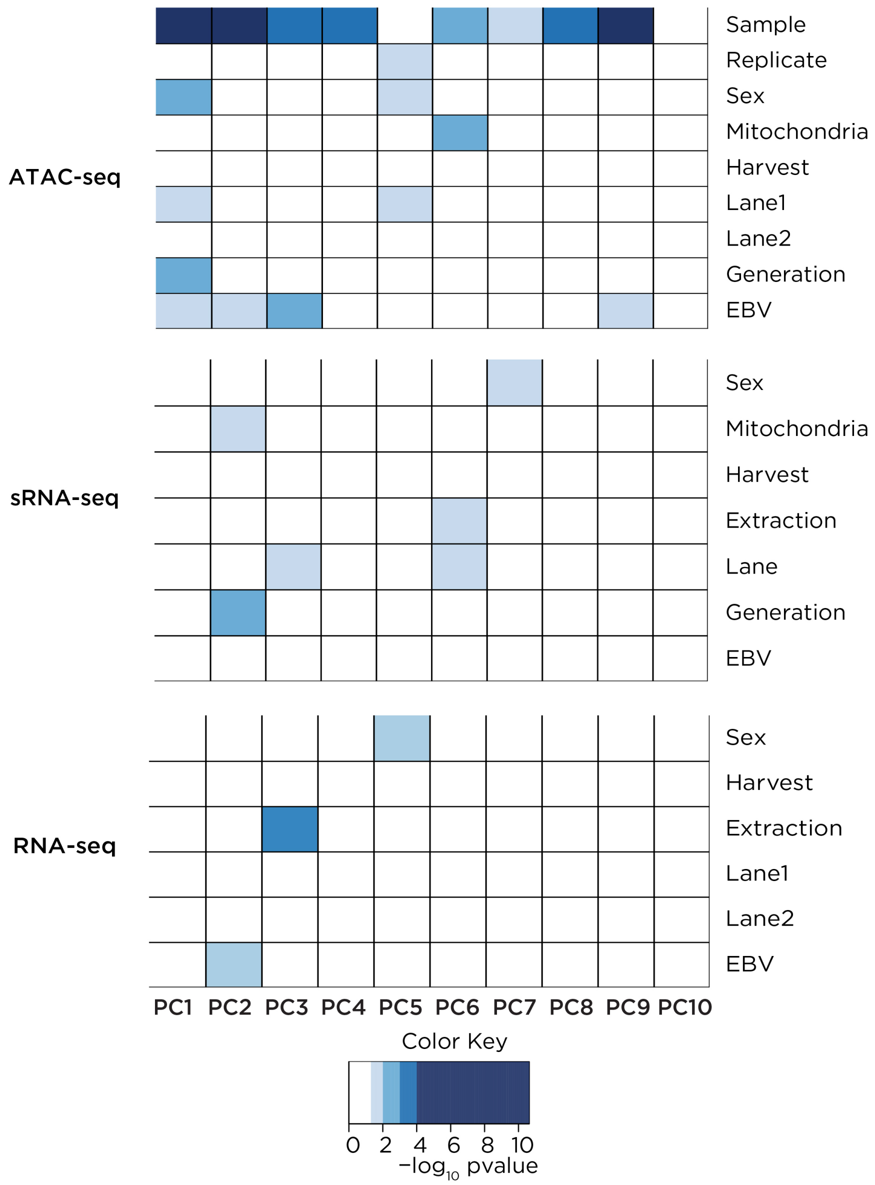

Figure S4: The sources of variability on our functional assays are shown using a heat map of linear regression of quantitative metadata against principal components capturing variability of each assay. These results informed how we adjusted our final results using linear modelling to remove unwanted sources of variability.

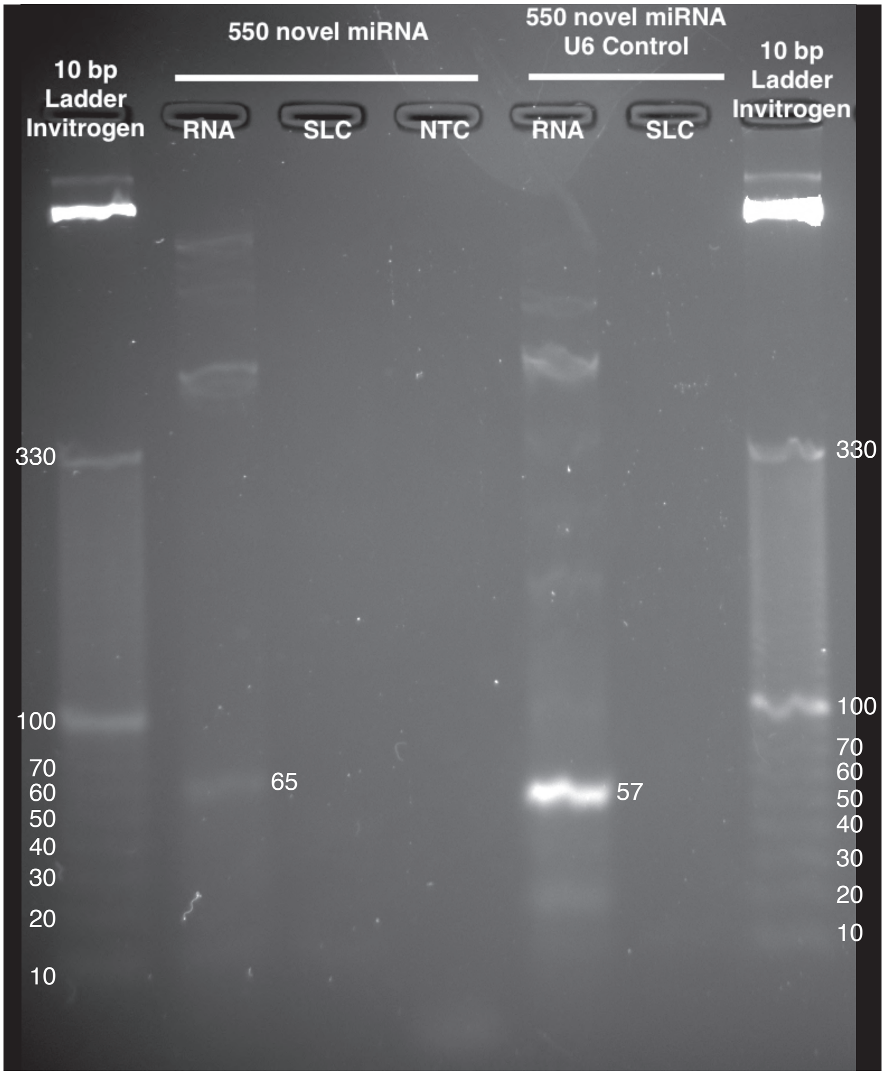

Figure S5: One novel microRNA was tested and confirmed using stem-loop PCR. A control U6 snRNA is shown to amplify on the right at its expected fragment length of 57 bp, while our novel miRNA amplifies at its expected fragment length of 65 bp on the left. SLC: stem-loop control, omits RNA template but keeps stem-loop primer; NTC: no template control, also omits RNA template as well as stem-loop primer.

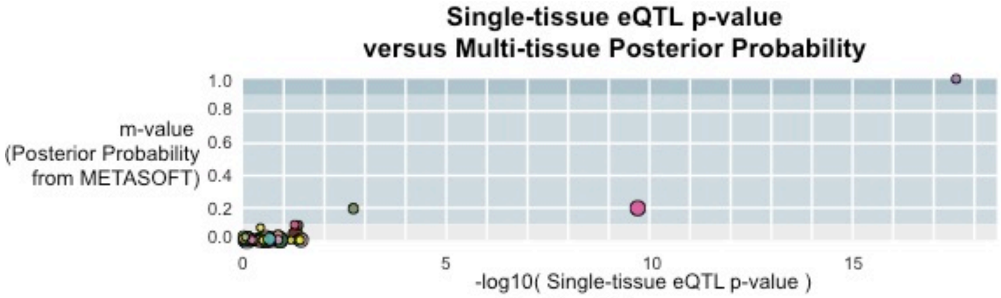

Significant Tissues

Tissue	Samples	NES	p-value	m-value
LCLs	117	0.963	2.8e-18	1.00
Spleen	146	0.217	1.9e-3	0.195
Vagina	106	0.213	0.05	0.0940
Whole Blood	369	0.142	1.9e-10	0.198

Nonsignificant Tissues

- Brain - Substantia nigra
- Small Intestine - Terminal Ileum
- Esophagus - Mucosa
- Artery - Coronary
- Breast - Mammary Tissue
- Artery - Aorta
- Brain - Putamen (basal ganglia)
- Brain - Hypothalamus
- Pituitary
- Thyroid
- Skin - Not Sun Exposed (Suprapubic)
- Skin - Sun Exposed (Lower leg)
- Uterus
- Nerve - Tibial
- Adipose - Subcutaneous
- Colon - Transverse
- Lung
- Brain - Cerebellar Hemisphere
- Heart - Atrial Appendage
- Prostate
- Pancreas
- Stomach
- Heart - Left Ventricle
- Adrenal Gland
- Colon - Sigmoid
- Muscle - Skeletal
- Testis
- Minor Salivary Gland
- Brain - Amygdala
- Artery - Tibial
- Cells - Transformed fibroblasts
- Adipose - Visceral (Omentum)
- Brain - Hippocampus
- Brain - Nucleus accumbens
- Brain - Cortex
- Brain - Cerebellum
- Liver
- Brain - Anterior cingulate cortex (BA24)
- Esophagus - Muscularis
- Ovary
- Brain - Caudate (basal ganglia)
- Brain - Frontal Cortex (BA9)
- Brain - Spinal cord (cervical c-1)
- Esophagus - Gastroesophageal Junction

Figure S6: Data from the GTEx project show that the intronic enhancer in *TBC1D4* is especially likely to act as an eQTL in LCLs, prompting our decision to use this cell type for genetic and epigenetic editing using CRISPR.

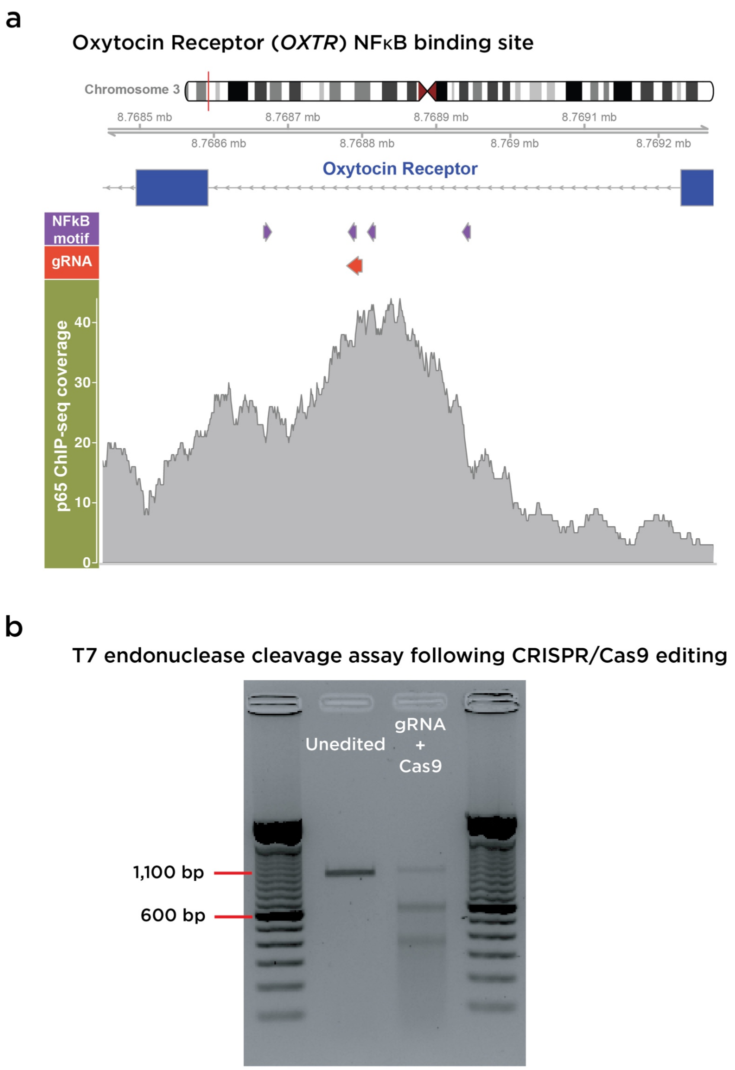

Figure S7: The Oxytocin Receptor *OXTR* gene has a strong intronic binding site for p65 (a). We tested whether our guide RNA (red arrow) was efficiently targeting this locus by performing CRISPR/Cas9 editing, amplifying the locus by PCR and using the T7 endonuclease cleavage assay to see the extent of cleavage at the locus due to sequence mismatches produced by Cas9. The decrease in intensity of the ~1,100 bp band in the lane from the edited cells is substantial, indicating efficient targeting of the locus by our guide RNA.
