# Supplementary material for: Functional genetic variants can mediate their regulatory effects through alteration of transcription factor binding
Source: Nat Commun. 2019 Aug 2;10:3472. doi: 10.1038/s41467-019-11412-5 (PMC6677801; doi:10.1038/s41467-019-11412-5)
Supplement: Supplementary file 3 — Description of Additional Supplementary Files [file 41467_2019_11412_MOESM3_ESM.pdf]

Description of Additional Supplementary Files

File name: Supplementary Data 1

Description: eGenes in common between the current study and that of Li, X. et al. Am. J. Hum. Genet. 95, 245–256 (2014).

File name: Supplementary Data 2

Description: qRT-PCR primers and results of validation of eGenes.

File name: Supplementary Data 3

Description: Gene and miRNA targets of eQTLs and summit locations for chromatin accessibility QTLs.

File name: Supplementary Data 4

Description: ATAC-seq peaks and summit locations called by combining data from all 17 family members.

File name: Supplementary Data 5

Description: Transcription factor motifs found to be enriched in ATAC-seq peaks shared by multiple individuals in the family studied.

File name: Supplementary Data 6

Description: DNA methylation values and coverage data for CGs located in the immediate vicinity of the *TBC1D4* multifunctional variant.

File name: Supplementary Data 7

Description: Metrics of sequencing performed for ATAC-seq, RNA-seq, whole genome bisulphite sequencing (WGBS) and small RNA-seq (sRNA-seq).

File name: Supplementary Data 8

Description: Oligonucleotides used in PCR, cloning and genomic editing experiments.
